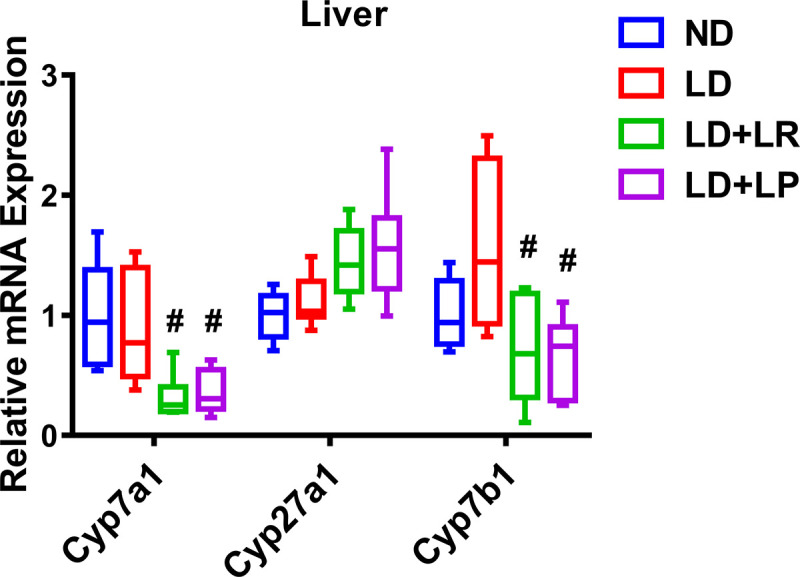# Correction for Ye et al., “FXR Signaling-Mediated Bile Acid Metabolism Is Critical for Alleviation of Cholesterol Gallstones by *Lactobacillus* Strains”

**DOI:** 10.1128/spectrum.05072-22

**Published:** 2023-02-01

**Authors:** Xin Ye, Dan Huang, Zhixia Dong, Xiaoxin Wang, Min Ning, Jie Xia, Shuang Shen, Shan Wu, Yan Shi, Jingjing Wang, Xinjian Wan

## AUTHOR CORRECTION

Volume 10, no. 5, e00518-22, 2022, https://doi.org/10.1128/spectrum.00518-22. Page 8, Fig. 3: Panel G is an inadvertent duplication of the graph in panel H. The correct panel G is shown below. The duplication had no impact on the conclusions of our paper.[Fig fig3]

**Figure fig3:**