# Supplementary material for: FXR Signaling-Mediated Bile Acid Metabolism Is Critical for Alleviation of Cholesterol Gallstones by Lactobacillus Strains
Source: Microbiol Spectr. 2022 Aug 29;10(5):e00518-22. doi: 10.1128/spectrum.00518-22 (PMC9603329; doi:10.1128/spectrum.00518-22)
Supplement: Supplemental file 1 — Supplemental material. Download spectrum.00518-22-s0001.pdf, PDF file, 1.3 MB [file spectrum.00518-22-s0001.pdf]

Supplementary Information

This supplementary information includes supplementary materials and methods, six supplementary figures and three supplementary table.

Supplementary Method

Oral Glucose Tolerance Test (OGTT)

After 6 h of food deprivation, the mice were orally administered a dose of 2.0 g/kg body weight glucose. Blood samples were collected from the tail at 0, 15, 30, 60 and 120 min after glucose administration, and blood glucose levels were measured with a blood glucose meter (Accu Check; Roche Diagnostics, Mannheim, Germany).

Supplementary Figures

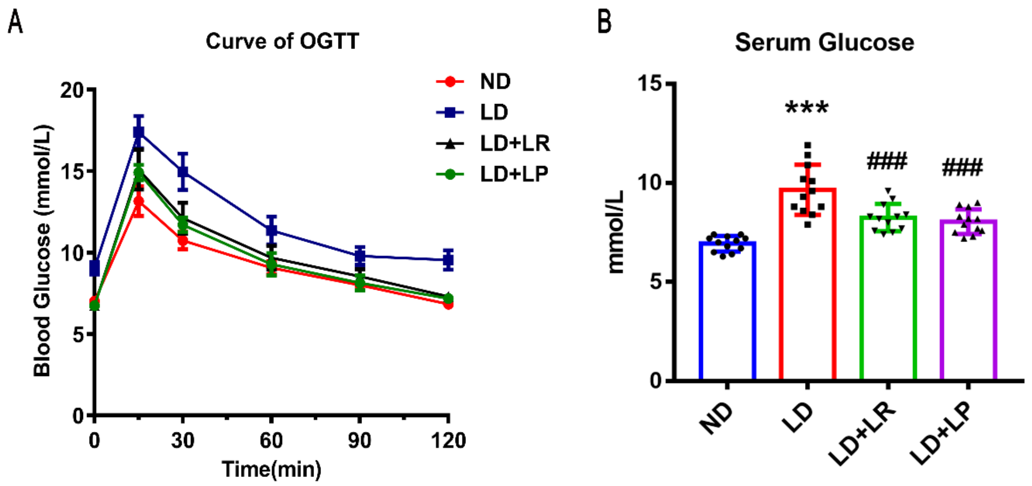

Supplementary Figure 1 LR and LP Treatments Reduced Glucose Metabolism Disorders in LD-fed Mice.

(A) OGTT curve. (B) Serum glucose was determined by a SIEMENS fast automatic

biochemical analyzer (ADVIA 2400). Data were analyzed by ANOVA along with a post-hoc Tukey's test and presented as the mean ± SEM (n = 12), * $0.01 < P \leq 0.05$, ** $0.001 < P \leq 0.01$, *** $P \leq 0.001$ vs ND, # $0.01 < P \leq 0.05$, ## $0.001 < P \leq 0.01$, ### $P \leq 0.001$ vs LD.

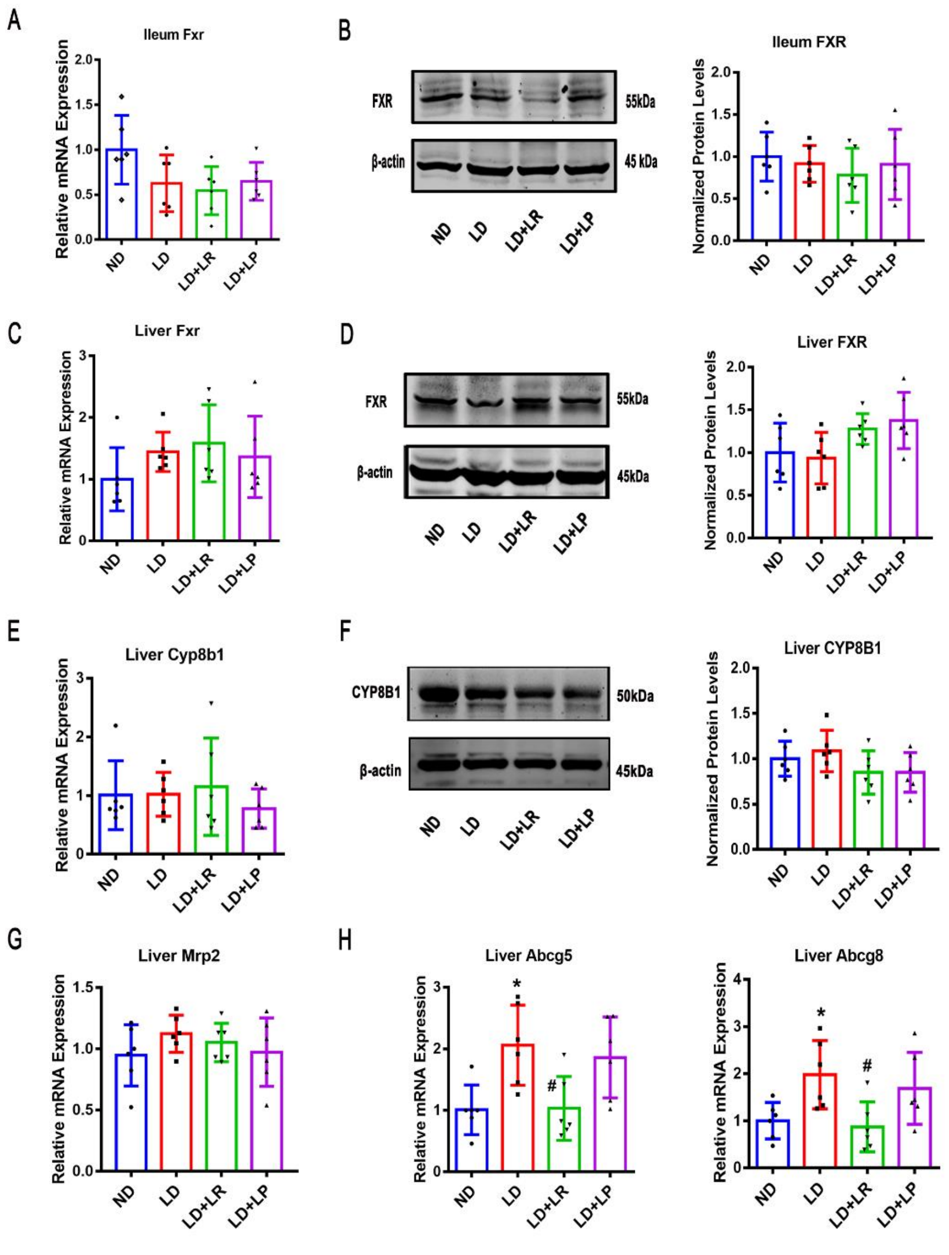

Supplementary Figure 2 LR and LP Treatments Activated FXR Signaling Pathway

and Cholesterol Transportation.

(A) Ileal mRNA expression of Fxr. (B) Protein expression and quantification of ileal FXR. (C) Hepatic mRNA expression of Fxr. (D) Protein expression and quantification of Hepatic FXR. (E) Hepatic mRNA expression of Cyp8b1. (F) Protein expression and quantification of Hepatic CYP8B1. (G-H) Hepatic mRNA expression of Mrp2, Abcg5, Abcg8. Data were analyzed by ANOVA along with a post-hoc Tukey's test and presented as the mean ± SEM (n=6), * $0.01 < P \leq 0.05$, ** $0.001 < P \leq 0.01$, *** $P \leq 0.001$ vs ND, # $0.01 < P \leq 0.05$, ## $0.001 < P \leq 0.01$, ### $P \leq 0.001$ vs LD.

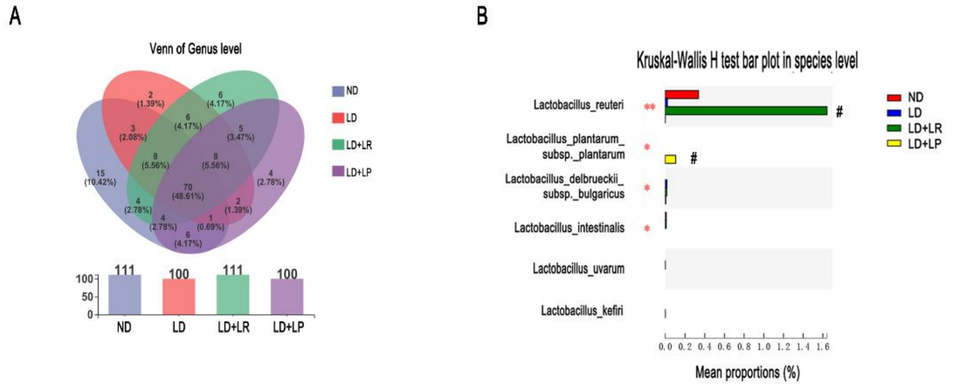

Supplementary Figure 3 LR and LP Treatments Changed the CGS-associated Gut Microbiota Composition in LD-fed Mice.

(A) Venn diagram of gut microbiota at the genus level modulated by the two probiotics LR and LP. (B) Comparison of different species of *Lactobacillus* abundance in the ND, LD, LD+LR and LD+LP groups by Kruskal-Wallis H test with the post-hoc (Tukey-Kramer) analysis. (n=5, # $0.01 < P \leq 0.05$, ## $0.001 < P \leq 0.01$, ### $P \leq 0.001$ vs LD. The red * in figure 4F showed statistically significant differences between the groups

by Kruskal-Wallis H test. * 0.01 < $P \leq 0.05$, ** 0.001 < $P \leq 0.01$, *** $P \leq 0.001$).

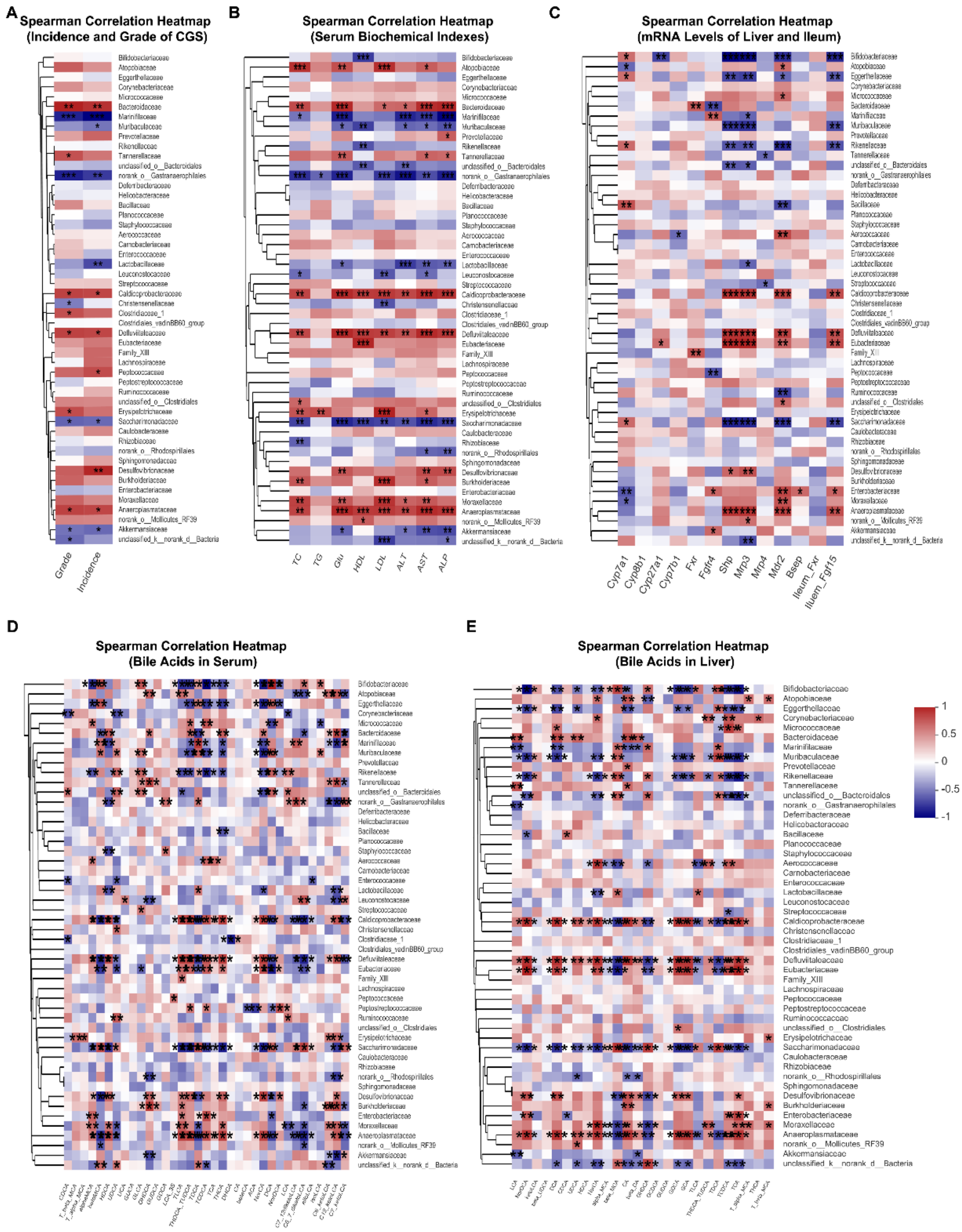

Supplementary Figure 4 Correlation between Gut Microbiota and Host CGS-related Parameters.

(A) Spearman’s correlation analysis was performed between the top 50 most abundant

families and the incidence and grade of CGS. (B) Spearman's correlation analysis was performed between the top 50 most abundant families and serum AST, ALT, ALP, TG, TC, HDL, LDL and glucose (Glu). (C) Spearman's correlation analysis was performed between the top 50 most abundant families and the mRNA levels of liver Cyp7a1, Cyp8b1, Cyp7b1, Cyp 27a1, Mdr2, Mrp3, Mrp4, Bsep, Shp, Fxr, and Fgfr4 and ileum Fxr, Fgf15, and Shp. (D) Spearman's correlation analysis was performed between the top 50 most abundant family levels and 35 kinds of BAs in the serum of mice. (E) Spearman's correlation analysis was performed between the top 50 most abundant family levels and 26 kinds of BAs in the livers of mice. Red and blue denote positive and negative associations, respectively. Red and blue denote positive and negative associations, respectively. (* 0.01 < $P \leq 0.05$, ** 0.001 < $P \leq 0.01$, *** $P \leq 0.001$, $|R| \geq 0.5$).

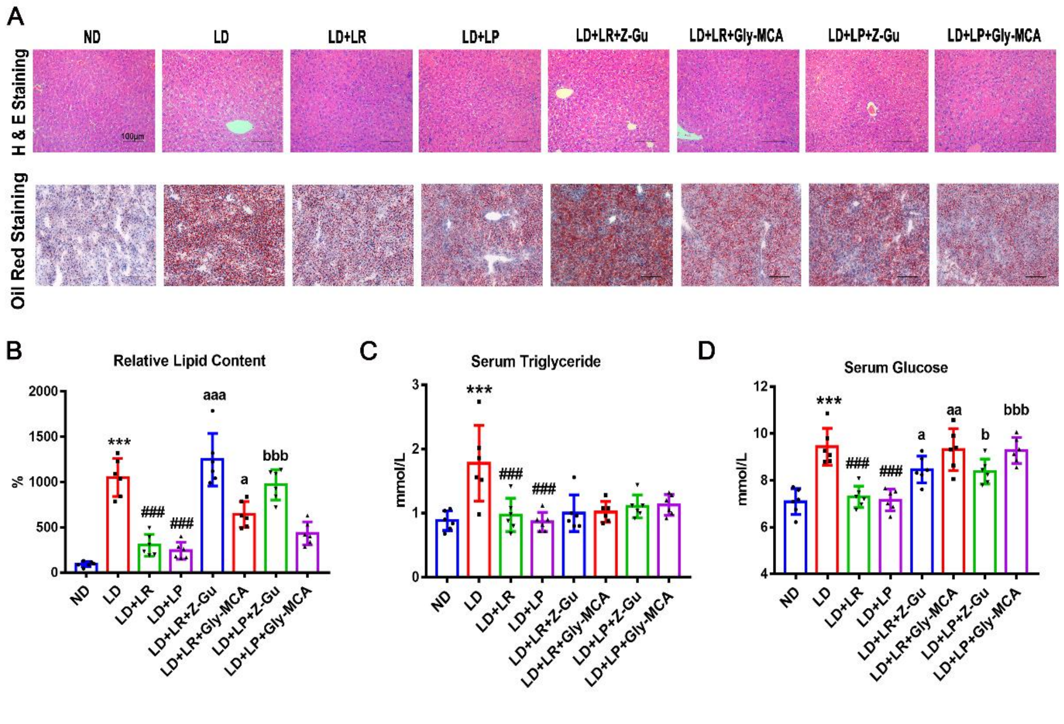

Supplementary Figure 5 The FXR Inhibitor Blocked the Improvements of Hepatic Steatosis and Serum Glucose Disorder Induced by LR and LP.

(A) Representative images of H&E-stained and Oil red O-stained liver sections (×200) (B) Percentage of Oil Red O positive area. (C) Serum triglyceride and glucose was determined by a SIEMENS fast automatic biochemical analyzer (ADVIA 2400). Data were analyzed by ANOVA along with a post-hoc Tukey's test and presented as the mean ± SEM (n=6), * $0.01 < P \leq 0.05$, ** $0.001 < P \leq 0.01$, *** $P \leq 0.001$ vs ND, # $0.01 < P \leq 0.05$, ## $0.001 < P \leq 0.01$, ### $P \leq 0.001$ vs LD, **a** $0.01 < P \leq 0.05$, **aa** $0.001 < P \leq 0.01$, **aaa** $P \leq 0.001$ vs LD+LR, **b** $0.01 < P \leq 0.05$, **bb** $0.001 < P \leq 0.01$, **bbb** $P \leq 0.001$ vs LD+LP.

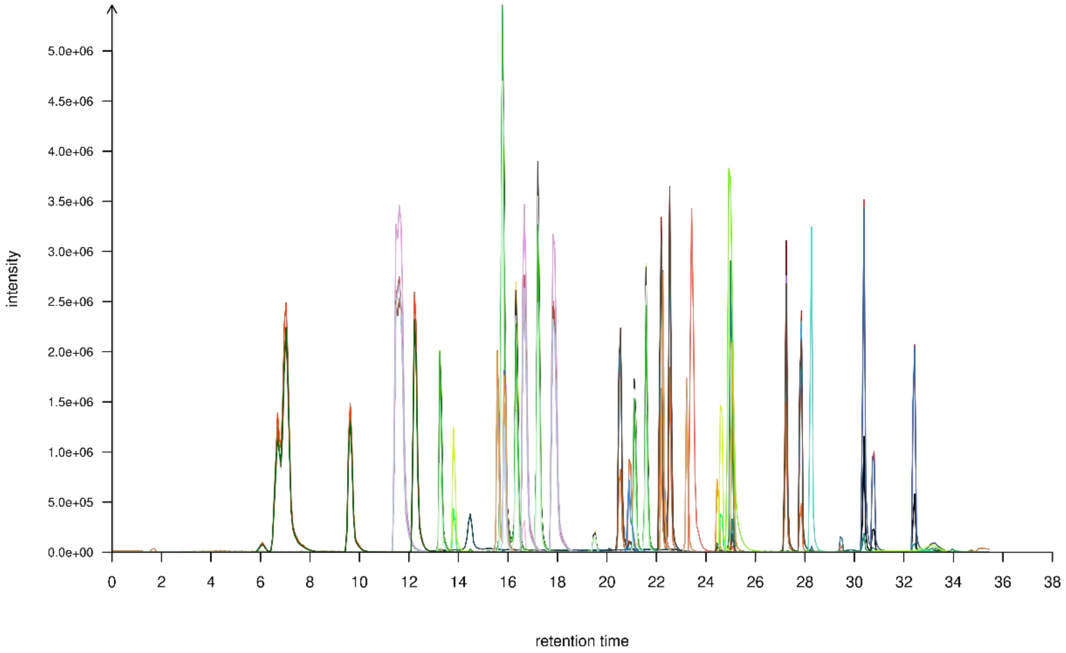

Supplementary Figure 6 The Total Ion Chromatography (TIC) of Working Standard Solutions.

Supplementary Table 1 PCR Genes Primers Sequences

Gene (mouse)		Primer sequences
Mrp2	Forward	5’- GCTTCCCATGGTGATCTCTT -3’
	Reverse	5’- ATCATCGCTTCCCAGGTACT -3’
Abcg5	Forward	5’- AGACGTTGCGATACACAGCGATG -3’
	Reverse	5’- GTGCCACAGAACACCAACTCTCC -3’
Abcg8	Forward	5’- CTCGTGTGGTTGGTGGTCTTCTG -3’
	Reverse	5’- GCCGTAGCTGATGCCGATGAC -3’
Fxr	Forward	5’- AGCATTACCAAGAACGCCGTGTAC -3’
	Reverse	5’- GCTGTCGTCCTCATTAGCTGTCTG -3’
Cyp8b1	Forward	5’- GGCAAGAAGATCCACCACTACAGC -3’
	Reverse	5’- TCAGGCGATAGAGGAAGCGTACC-3’

Supplementary Table 2 Standard Curve Concentration of Each Bile Acid

Name（ng/mL）	Cal1	Cal2	Cal3	Cal4	Cal5	Cal6	Cal7	Cal8	Cal9	Cal10
alloLCA	800	400	200	100	50	25	10	5	2.5	1
LCA	400	200	100	50	25	12.5	5	2.5	1.25	0.5
isoLCA	400	200	100	50	25	12.5	5	2.5	1.25	0.5
NorDCA	200	100	50	25	12.5	6.25	2.5	1.25	0.625	0.25
6-ketoLCA	800	400	200	100	50	25	10	5	2.5	1
12-ketoLCA	1000	500	250	125	62.5	31.25	12.5	6.25	3.125	1.25

7-ketoLCA	200	100	50	25	12.5	6.25	2.5	1.25	0.625	0.25
β-UDCA	400	200	100	50	25	12.5	5	2.5	1.25	0.5
DCA	400	200	100	50	25	12.5	5	2.5	1.25	0.5
CDCA	400	200	100	50	25	12.5	5	2.5	1.25	0.5
UDCA	200	100	50	25	12.5	6.25	2.5	1.25	0.625	0.25
HDCA	1000	500	250	125	62.5	31.25	12.5	6.25	3.125	1.25
NorCA	800	400	200	100	50	25	10	5	2.5	1
DHCA	800	400	200	100	50	25	10	5	2.5	1
7,12-diketo LCA	800	400	200	100	50	25	10	5	2.5	1
6,7-diketo LCA	800	400	200	100	50	25	10	5	2.5	1
α-MCA	1000	500	250	125	62.5	31.25	12.5	6.25	3.125	1.25
UCA	200	100	50	25	12.5	6.25	2.5	1.25	0.625	0.25
β-MCA	1000	500	250	125	62.5	31.25	12.5	6.25	3.125	1.25
CA	400	200	100	50	25	12.5	5	2.5	1.25	0.5
ACA	200	100	50	25	12.5	6.25	2.5	1.25	0.625	0.25
βCA	2000	1000	500	250	125	62.5	25	12.5	6.25	2.5
GLCA	800	400	200	100	50	25	10	5	2.5	1
GHDCA	800	400	200	100	50	25	10	5	2.5	1
GCDCA	1000	500	250	125	62.5	31.25	12.5	6.25	3.125	1.25
GUDCA	800	400	200	100	50	25	10	5	2.5	1
GDCA	800	400	200	100	50	25	10	5	2.5	1
LCA-3S	800	400	200	100	50	25	10	5	2.5	1
GCA	1000	500	250	125	62.5	31.25	12.5	6.25	3.125	1.25

TLCA	800	400	200	100	50	25	10	5	2.5	1
THDCA	2000	1000	500	250	125	62.5	25	12.5	6.25	2.5
TUDCA	2000	1000	500	250	125	62.5	25	12.5	6.25	2.5
TDCA	1000	500	250	125	62.5	31.25	12.5	6.25	3.125	1.25
TCDCA	1000	500	250	125	62.5	31.25	12.5	6.25	3.125	1.25
TCA	1000	500	250	125	62.5	31.25	12.5	6.25	3.125	1.25
T-α-MCA	1000	500	250	125	62.5	31.25	12.5	6.25	3.125	1.25
THCA	400	200	100	50	25	12.5	5	2.5	1.25	0.5
T-β-MCA	1000	500	250	125	62.5	31.25	12.5	6.25	3.125	1.25
CDCA-G	3200	1600	800	400	200	100	40	20	10	5
GUCA	200	100	50	25	20	10	5	2.5	2	1

Supplementary Table 3 Detailed Information of Each Bile Acid

Name	Full Name	CAS #	Supplier #	InChI Key	Retention Time (min)	Precursor Ion	Product Ion
alloLCA	Allolithocholic acid	2276-93-9	B27763	SMEROWZSTRWXGI-NWFSOSCSSA-N	29.92	375.145	375.145
LCA	Lithocholic acid	434-13-9	B28100	SMEROWZSTRWXGI-HVATVPOCSA-N	31.92	375.3	375.3
isoLCA	Isolithocholic acid	1534-35-6	B65766	SMEROWZSTRWXGI-WFVDQZAMSA-N	30.32	375.301	375.301
NorDCA	23-Nordeoxycholic acid	53608-86-9	B74559	PLRQOCVIINWCFA-AHFDLSHQSA-N	24.36	377.3	377.3
6-ketoLCA	6-ketolithocholic acid	106439-47-8	B79566	JWZBXKZZDYMDCJ-IJPFKRJSSA-N	28.99	389.238	389.238

12-ketoLCA	12-ketolithocholic acid	5130-29-0	S22145	CVNYHSDFZXHMMJ-VPUMZWJWSA-N	24.42	389.301	389.301
7-ketoLCA	7-ketolithocholic acid	4651-67-6	B26465	DXOCDBGWDZAYRQ-AURDAFMXSA-N	23.79	389.302	389.302
β-UDCA	3β-Ursodeoxycholic acid	78919-26-3	B72964	RUDATBOHQWOJDD-YAKZLEHISA-N	19.65	391.254	391.254
DCA	Deoxycholic acid	83-44-3	B21032	KXGVEGMKQFWNSR-LLQZFEROSA-N	27.32	391.3	391.3
CDCA	Chenodeoxycholic acid	474-25-9	B20347	RUDATBOHQWOJDD-BSWAIDMHSA-N	26.69	391.301	391.301
UDCA	Ursodeoxycholic acid	128-13-2	B21405	RUDATBOHQWOJDD-UZVSRGJWSA-N	21.43	391.302	391.302
HDCA	Hyodeoxycholic acid	83-49-8	B21672	DGABKXLVXPYZII-SIBKNCMHSA-N	21.83	391.303	391.303
NorCA	Norcholic acid	60696-62-0	B74560	SHUYNJFEXPRUGR-RTCCEZQESA-N	15.95	393.211	329.1
DHCA	Dehydrocholic acid	81-23-2	B24725	OHXPGWPVLFPUSM-KLRNGDHRSA-N	13.78	401.2	401.2
7,12-diketo LCA	7,12-diketolithocholic acid	517-33-9	B27766	MAFJMPFLJJCSTB-FQBQTYDJSA-N	13.16	403.14	403.14
6,7-diketo LCA	6,7-diketolithocholic acid	N/A	S40141	FRIRHJVKXFYECW-ZTERCDMUSA-N	23.95	403.3	403.3
α-MCA	α-Muricholic acid	2393-58-0	S22142	DKPMWHFRUGMUKF-GDYCBZMLSA-N	15.68	407.3	407.3
UCA	Ursocholic acid	2955-27-3	B22360	BHQCQFFYRZLCQQ-UTLSPDKDSA-N	12.64	407.301	407.301

β-MCA	β-Muricholic acid	2393-59-1	B74541	DKPMWHFRUGMUKF-CRKPLTDNSA-N	16.46	407.302	407.302
CA	Cholic acid	81-25-4	B20274	BHQCQFFYRZLCQQ-OELDTZBJSA-N	20.81	407.303	407.303
ACA	Allocholic acid	2464-18-8	B22337	BHQCQFFYRZLCQQ-PGHAKIONSA-N	20.33	407.304	407.304
βCA	3β-Cholic acid	3338-16-7	T51018	BHQCQFFYRZLCQQ-UXWVVXDJSA-N	15.13	407.362	407.362
GLCA	Glycolithocholic acid Sodium Salt	24404-83-9	B74545	LQKBJAKZKFBLIB-LGURPPGFSA-M	27.83	432.401	73.9
GHDCA	Glycohyodeoxycholic acid	13042-33-6	B27133	SPOIYSFQOFYOFZ-BRDORRHWSA-N	15.28	448.2	74
GCDCA	Glycochenodeoxycholic acid Sodium Salt	16564-43-5	S31335	AAYACJGHNRIFCT-YRJJIGPTSA-M	21.59	448.276	73.9
GUDCA	Glycoursodeoxycholic acid	64480-66-6	B32965	GHCZAUBVMUEKKP-XROMFQGDSA-N	15	448.277	73.9
GDCA	Glycodeoxycholic acid Sodium Salt	16409-34-0	B27134	VMSNAUAEKXEYGP-YEUHZSMFSA-M	22.63	448.279	73.9
LCA-3S	Lithocholic acid 3-sulfate	64936-81-8	T70708	AXDXVEYHEODSPN-HVATVPOCSA-N	26.59	455.196	96.9
GCA	Sodium Glycocholate Hydrate	338950-81-5	S31334	YWROUPFMHKARON-HJRQWJHVSA-M	15.27	464.281	73.9
TLCA	Taurolithocholic acid Sodium Salt	6042-32-6	S26225	YAERYJYXPRIDTO-HRHHVWJRSA-M	24.72	482.223	80
THDCA	Taurohyodeoxycholic Acid	110026-03-4	B27364	HMXPOCDLAFAFNT-BHYUGXBJSA-N	12.22	498.224	79.9

TUDCA	Tauroursodeoxycholic Acid	14605-22-2	B20921	BHTRKEVKTKCXOH-LBSADWJPSA-N	12.22	498.25	80
TDCA	Taurodeoxycholic acid Sodium Salt	1180-95-6	S30708	YXHRQQJFKOHLAP-FVCKGWAHSA-M	19.1	498.35	79.8
TCDCA	Taurochenodeoxycholic acid	516-35-8	B20919	BHTRKEVKTKCXOH-BJLOMENOSA-N	17.68	498.357	79.8
TCA	Taurocholic acid Sodium Salt	145-42-6	B20918	JAJWGJBVLPIOOH-QGRZANFFSA-M	12.9	514.332	79.8
T-α-MCA	Tauro-α-muricholic acid	25613-05-2	B74540	XSOLDPYUICCHJX-UHFFFAOYSA-N	7.41	514.337	79.9
THCA	Taurohyocholic acid	32747-07-2	YY80220	XSOLDPYUICCHJX-QZEPYOAJSA-N	10.25	514.343	79.9
T-β-MCA	Tauro-β-muricholic acid	25696-60-0	B27767	XSOLDPYUICCHJX-OEYGYFRSSA-N	7.74	514.346	79.9
CDCA-G	Chenodeoxycholic acid 24-Acyl-β-D-glucuronide	208038-27-1	T48407	ZTJBLIAPAIPNJE-BWGRGVIUSA-N	20.33	567.525	391.1
GUCA	Glycoursocholic acid	95093-95-1	YY91138	RFDAIACWWDREDC-IFZPJRIXSA-N	36.74	416.2	73.9
